# G-CSF promotes the development of hepatocellular carcinoma by activating the PI3K/AKT/mTOR pathway in TAM

**DOI:** 10.18632/aging.205922

**Published:** 2024-07-01

**Authors:** Heng Cao, Shunxiang Wang

**Affiliations:** 1 Department of Hepatobiliary Surgery, The Fourth Hospital of Hebei Medical University, Shijiazhuang, Hebei 050011, P.R. China

**Keywords:** hepatocellular carcinoma, granulocyte colony-stimulating factor, PI3K/AKT/mTOR signaling pathway, tumor-associated macrophages

## Abstract

Objective: This investigation seeks to elucidate the role of the Granulocyte Colony-Stimulating Factor (G-CSF) in the progression of hepatocellular carcinoma (HCC), as well as the impact of the substance on related signaling pathways within the disease matrix.

Methods: Nude mouse tumor-bearing assay was used to detect tumor progression. Levels of Mannose/CD68 and CD34/Mannose within these samples and the concentrations of Mannose and inducible Nitric Oxide Synthase (iNOS) in macrophages were quantified using immunofluorescence techniques. The angiogenic capability was assessed via tube formation assays, and protein expressions of G-CSF, Vascular Endothelial Growth Factor (VEGF), Transforming Growth Factor-beta (TGF-β), Matrix Metalloproteinases 2 and 9 (MMP2/9), SH2-containing protein tyrosine phosphatase-2 (SHP-2), phosphorylated PI3K/total PI3K (P-PI3K/t-PI3K), phosphorylated AKT/total AKT (P-AKT/t-AKT), and phosphorylated mTOR/total mTOR (P-mTOR/t-mTOR) were measured through Western Blot analysis in both tumor tissues and macrophages.

Results: Administration of G-CSF resulted in a marked augmentation of tumor volume. Macrophage Mannose expression was significantly elevated upon G-CSF treatment, while iNOS levels were conspicuously diminished. G-CSF substantially enhanced the secretion of VEGF, TGF-β, and MMPs in tumor tissues. Macrophage parameters, following incubation in G-CSF pre-treated conditioned medium, indicated enhanced tube-forming capabilities relative to the control, an effect mitigated by the introduction of specific inhibitors. Furthermore, the G-CSF group exhibited a notable reduction in SHP-2 expression, alongside a substantial elevation in the phosphorylation levels of the PI3K/AKT/mTOR pathway proteins across all tumor-bearing paradigms.

Conclusion: G-CSF ostensibly facilitates the advancement of hepatocellular carcinoma by activating the PI3K/AKT/mTOR signaling cascade within Tumor-Associated Macrophages (TAM).

## INTRODUCTION

Hepatocellular carcinoma (HCC) emerges as a prevalent neoplasm within the alimentary system, ranking with grim morbidity and mortality that trails only behind lung, colorectal, and gastric cancers. Prominent etiological factors contributing to its inception include hepatitis B virus (HBV) infections, ethanolic hepatitis, and the consumption of aflatoxin-laden substances. Historically, treatments have fixated on direct obliteration of neoplastic cells. However, burgeoning research on neoplastic microenvironments marks a paradigm shift towards innovative prophylactic and therapeutic strategies. The tumoral microenvironment, as a haven for neoplastic cells, substantially fosters tumorigenesis and metastasis by precipitating angiogenesis, thwarting immunological vigilance, and dampening immune responses [1–3].

Advancements in understanding the tumoral microenvironment reveal that neoplastic cells have the propensity to enlist macrophages through the secretion of signaling molecules. In this metabolically distinctive milieu, a torrent of growth factors and proteolytic enzymes, nurtured by persistent immune responses, define its biological tenor. This environment is replete with inflammatory cells, particularly macrophages, which predominantly manifest phenotypically as M2-type within tumor-associated macrophages (TAMs), thereby aiding in the invasive assault on tissues, and vasculature, and enabling the remote spread of malignancy. The tumor microenvironment (TME) houses an array of cellular constituents; paramount among these are endothelial cells, integral to tumoral development, and the vascular networks created by these cells [4] provision tumor cells with requisite nutrients while concurrently expunging metabolic detritus, sustaining the robust metabolic needs of the neoplastic cells. The immune cell contingent, encompassing neutrophils, lymphocytes, and macrophages [4–6], is highlighted by macrophages [6] which, due to their adaptable phenotype, can be co-opted by tumors to promote macrophage polarization, thereby altering the local habitat to their proliferative advantage. It is documented that granulocyte-colony stimulating factor (G-CSF) can exert direct influence upon human monocytes, modulating cytokine secretions [7], and facilitating the phenotypic shift of macrophages from M1 to M2.

Granulocyte-colony stimulating factor (G-CSF), a polypeptide glycoprotein synthesized by the osseous marrow stromal cells, chiefly targets the lineage of bone marrow neutrophils to oversee their proliferation, differentiation, and activation and incites the amplification and liberation of neutrophils. In instances of neutropenia, G-CSF administration can burgeon neutrophil populations, hence serving a therapeutic function [5]. Diverse malignancies, including small cell lung carcinoma [6, 7], bladder, gallbladder [8, 9], and thyroid cancers [10] exhibit aberrant G-CSF expressions, though their expression in HCC is sparingly reported [11]. This 30KDa glycoprotein, a constituent of the hematopoietic growth factor family, acts selectively on granulocytic and macrophagic progenitor cells, to stimulate their expansion and maturation. Pioneering reports pinpointed a G-CSF-producing lung cancer, showcasing the anomalous G-CSF expressions in neoplastic entities [11]. Yet, elevated G-CSF expressions have also been observed in select HCC cases [12].

The PI3K/AKT/mTOR signaling cascade is instrumental in orchestrating cellular functions inclusive of growth, proliferation, sustenance, and metabolic processes. In hepatocellular carcinoma, the PI3K pathway often undergoes aberrant activation due to PI3K gene mutations or irregularities in factors upstream of the pathway, precipitating the activation of AKT and mTOR which, in turn, stimulate growth, proliferation, and dissemination of neoplastic cells. Furthermore, this aberrant signaling may impede apoptotic pathways in tumor cells, thus expediting oncogenesis. Evidence suggests that G-CSF can modulate cellular characteristics such as proliferation, survival, and differentiation by triggering the PI3K/AKT/mTOR axis, while SHP2 has been identified as an inhibitor of this signaling pathway. SHP2 (Src homology 2-containing protein tyrosine phosphatase 2) assumes a pivotal role in the pathology of hepatocellular carcinoma. This tyrosine phosphatase is integral in modulating an array of cellular signaling cascades. Within the milieu of hepatocellular carcinoma, anomalous expression and activation patterns of SHP2 have been correlated with both the genesis and the advancement of neoplastic formations. Research indicates that SHP2 exhibits heightened enzymatic activity within the cellular confines of hepatocellular carcinoma, thereby influencing numerous critical signaling pathways. Further explorations have elucidated that SHP2 engages in complex interactions with a variety of receptor tyrosine kinases implicated in liver oncogenesis — prominently including EGFR (epidermal growth factor receptor), MET (hepatocyte growth factor receptor), and IGF-1R (insulin-like growth factor 1 receptor) — along with subordinate signaling entities encompassing RAS, ERK, and PI3K/AKT/mTOR. These intricate interplays and regulatory mechanisms are instrumental in facilitating unrestrained cellular proliferation, robust growth, incursive behavior, and metastatic dissemination.

At this juncture, the pathogenic mechanisms by which G-CSF, synthesized by HCC tissues, instigates oncogenesis remain enigmatic, and there is an imperative to ascertain efficacious therapeutic strategies. Our investigation delves into the impact of G-CSF on HCC and its underlying molecular framework through the lens of Tumor-Associated Macrophages (TAM), thereby illuminating novel therapeutic approaches and objectives for the clinical management of hepatocellular carcinoma.

## METHODS

### Animal

Nude mice of the BALB/c strain employed for this study were obtained from the vivarium of Hebei Medical University. The Institutional Animal Care and Use Committee of Hebei Medical University's Fourth Hospital sanctioned all related experimental protocols, ensuring adherence to established standards of animal care. These rodents resided within the Specific Pathogen-Free (SPF) confines of the aforementioned institution's Experimental Animal Center, benefiting from a controlled climate, regulated photoperiods, and autoclaved potables and nourishments.

### Formation of neoplasm in murine models

HepG2 cells, harvested during their logarithmic phase of expansion, were enzymatically dissociated. Subsequently, the cell population was normalized to a density of 1 × 10^6^ cells/ml, then suspended in a basal medium at a volume of 200 μl, and conserved at 4°C for imminent application. Anesthesia was administered to the mice using a vaporized anesthetic apparatus; hair on their abdomen was eradicated using a depilatory agent, and a sterile 1 cm incision was executed to unveil the hepatic organ. The previously prepared cellular concoction was then inoculated into the left hepatic lobe at 200 μl per murine subject. Post-injection, the hepatic organ was repositioned, cutaneous and subcutaneous layers sutured, and the subjects were maintained at a temperature of 42°C postoperatively, with normal alimentation resuming upon their revival from anesthesia.

Upon their regaining consciousness, the rodent subjects were allocated at random into either a control cohort or one treated with Granulocyte-Colony Stimulating Factor (G-CSF). The latter group received an intraperitoneal injection of recombinant G-CSF, dissolved in saline at a concentration of 150 μg/kg, totaling 1 ml per injection. Conversely, the control group received the equivalent volume of saline alone. After a six-week duration of customary care, eight subjects from each cluster were euthanized via hyperanesthetic means, permitting subsequent assessments of neoplastic diameters to discern any disparities between the two factions.

### Immunofluorescent techniques

The sections from paraffin-embedded specimens were sequentially deparaffinized and rehydrated. Subjected to high-pressure antigen retrieval within a citrate buffer solution at a 0.01 mol/L concentration, the sections were incubated under heat for half an hour, followed by an equilibration to ambient temperature. A Tris-buffered saline (TBS) rinse was conducted thrice, for intervals of five minutes each. Endogenous peroxidase activity within these samples was quelled via immersion in a freshly concocted 3% hydrogen peroxide solution for a quarter of an hour at room temperature, succeeded by another series of three TBS rinses. Tissue sections were then treated with a 2% bovine serum albumin (BSA) solution for a duration of two hours, to occlude native antibodies and diminish nonspecific staining artifacts. The sections were relieved of goat serum, desiccated at their periphery, and blanketed with an appropriate measure of antibodies against Mannose-6-Phosphate (1:450), CD68 (1:400), CD34 (1:200), and a diluent evenly, ensconced within a light-shielded moist chamber at 4°C overnight. On the following morning, the sections were reacclimated to room temperature for 30 minutes following their retrieval from refrigeration. After a series of TBS washes, secondary antibodies conjugated with Alexa Fluor^®^ 488 and Alexa Fluor^®^ 594 were applied at a dilution of 1:500, with the sections kept in the dark for 45 minutes at room temperature. A final set of washes with TBS, shielded from light, was performed before mounting the sections with an anti-fade reagent containing 4′,6-diamidino-2-phenylindole (DAPI). Fluorescent activities were subsequently observed and documented utilizing a fluorescence microscope.

Following the placing of 10 μl of culture medium and a circular coverslip within a 24-well plate, THP-1 cell density was adjusted to 1 × 10^4^ cells/ml, and 100 μl of this cell suspension was deposited onto each coverslip. After 4 hours of incubation, the volume in each well was augmented to 500 μl and left overnight in the cell incubator. Post incubation, TBS rinses prepared the cells for fixation in 10% neutral-buffered formalin for 15–20 minutes. The cover slips were cleansed thrice with TBS after fixation, and then rendered permeable via Triton X-100 treatment. Subsequent immunofluorescence protocol steps mirrored those detailed above, employing primary antibodies specific to Mannose-6-Phosphate (1:450), CD68 (1:400), inducible nitric oxide synthase (iNOS) (1:50), and secondary antibodies linked to Alexa Fluor^®^ dyes.

### Western blot analysis

Following macrophage culture and tissue homogenization, the protein was extracted by centrifugation under cold conditions (4°C). The protein samples, quantified as 20 μg each, were subjected to SDS-PAGE, commencing with the stack at 80V for 40 minutes and the resolving gel at 110V for 60 minutes. The separation enabled the transfer of the proteins onto polyvinylidene difluoride (PVDF) membranes, which were then blocked either with 5% non-fat milk powder or BSA. Membranes were incubated with primary antibodies directed against a spectrum of proteins, including G-CSF, Vascular Endothelial Growth Factor (VEGF), Transforming Growth Factor Beta (TGF-β), matrix metalloproteinases MMP2 and MMP9, Src homology 2-containing protein tyrosine phosphatase-2 (SHP-2), phosphoinositide 3-kinase (both total and phosphorylated), and Glyceraldehyde 3-Phosphate Dehydrogenase (GAPDH). After a trio of washes with Tris-buffered saline containing Tween 20 (TBST), the membranes were oscillated in a secondary antibody solution for 2 hours, then washed again, and finally subjected to chemiluminescent detection.

### Evaluation of cellular vitality via the cell counting Kit-8 methodology

The assessment of cellular vitality was conducted using the Cell Counting Kit-8 (CCK-8). The cellular specimens were incubated in ninety-six well microtiter plates over a duration of forty-eight hours, after which they were exposed to a 10-microliter volume of the CCK-8 reagent. This exposure lasted for a span of one hour at a temperature of 37 degrees Celsius within an ambient milieu enriched with 5% carbon dioxide and maintained at a relative humidity threshold of 95%. Thereafter, the optical density was ascertained at a spectral measurement of 450 nanometers, utilizing a microplate spectrophotometer.

### Angiogenesis assay

A co-culture of THP-1 and SVEC4-10 cells ensued, with SVEC4-10 cells adjusted to a density of 1 × 10^6^ cells/100 μl post-pancreatic enzyme digestion. Cell suspensions were allocated into 24-well plates, with the control group receiving fresh RPMI 1640 medium and the G-CSF group’s medium enriched with macrophage-conditioned supernatant. G-CSF fortification continued in the supplement medium. After 8-hour incubation, inverted microscopy allowed for image capture, focusing on key metrics like the total number of junctions and cumulative tube length. These images were subjected to quantitative analysis through the ImageJ Software suite, with random field selection.

### Statistical consideration

The application of Prism 9.0 software facilitated the statistical evaluation of the data, with mean and standard deviation characterizing the measurement data. A subsequent comparison of data, adherent to normal distribution and variance homogeneity, was executed employing an independent sample *t*-test. A *p*-value falling below the threshold of 0.05 was deemed as an indication of statistical significance.

## RESULTS

### G-CSF enhances hepatocellular carcinoma progression

**Figure 1:**
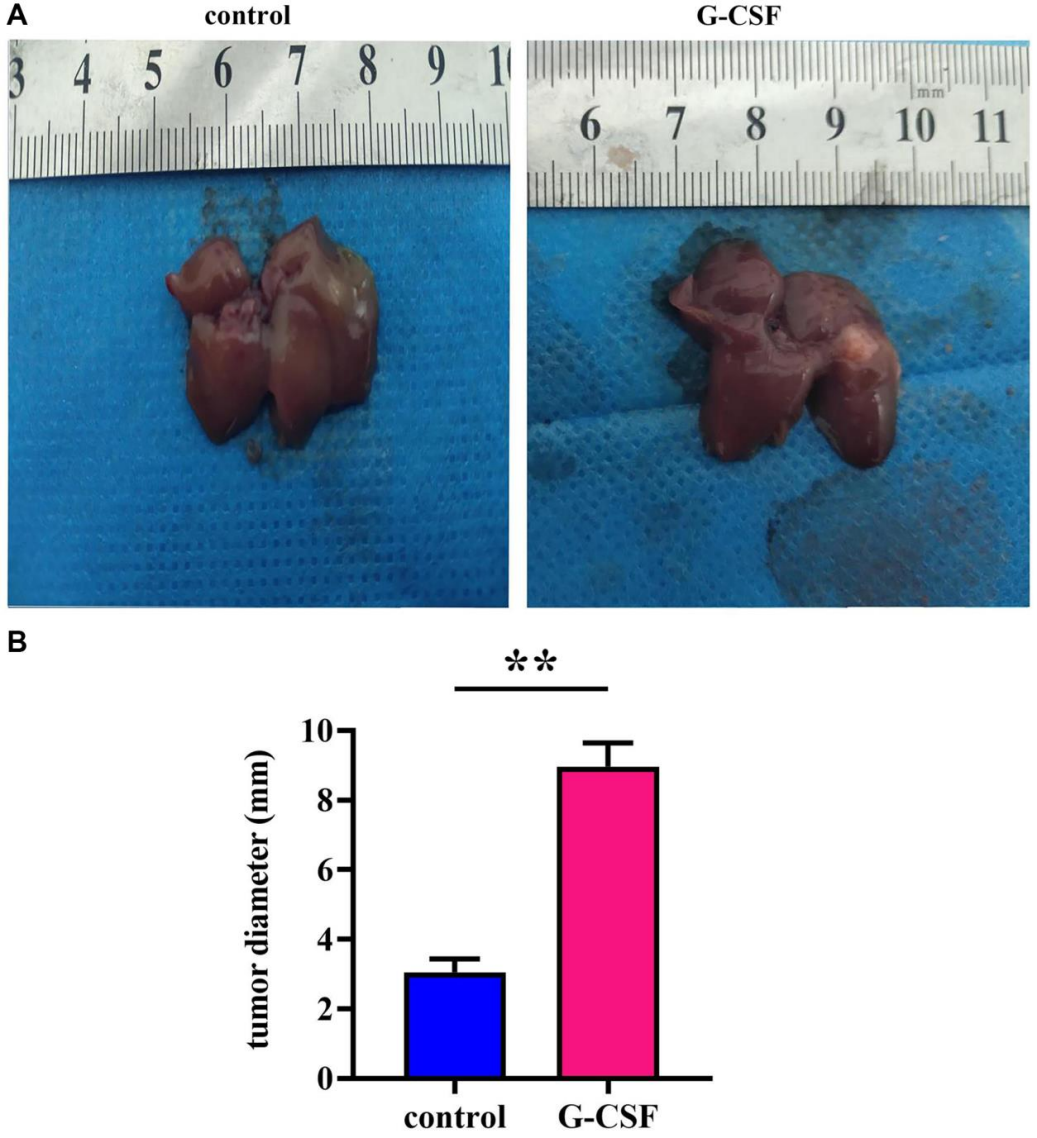
**Neoplastic engraftment experimentation in athymic rodents.** (**A**) Observations of intrinsically engrafted neoplasms in murine models. (**B**) Quantitative analyses of neoplastic diameters. The *P*-value is less than 0.05.

Granulocyte Colony-Stimulating Factor (G-CSF) has been observed to facilitate the proliferation of hepatocellular carcinoma, as detailed in experimental observations on nude mice bearing tumors. Notably, tumor diameters in the mice receiving G-CSF treatment were markedly enlarged in comparison to those in the control cohort (refer to Figure 1). These findings underscore the potential of G-CSF to enhance both the incidence and growth of hepatocellular carcinoma.

### G-CSF facilitates M2 macrophage polarization and subsequent hepatocellular carcinoma advancement

**Figure 2:**
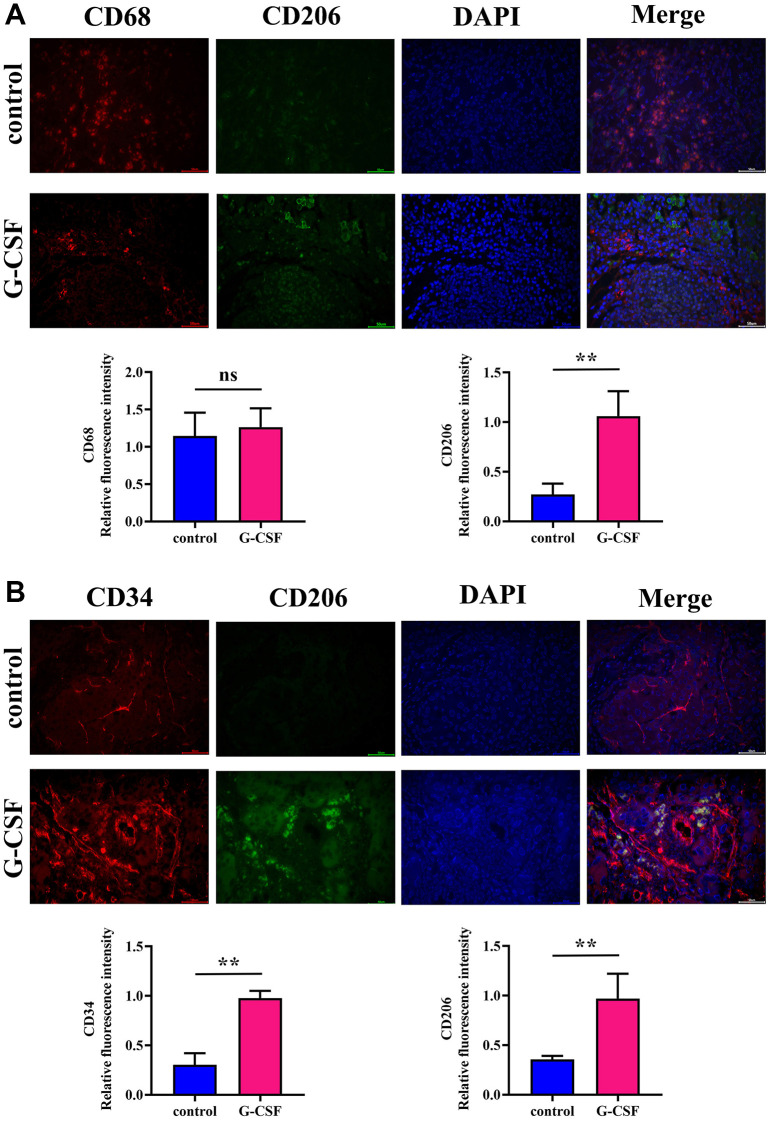
**Assessment of M2-type macrophage expression and angiogenic quantification in neoplastic tissues via immunofluorescence.** (**A**) Detection of Mannose (depicted in green)/CD68 (depicted in red) expression levels within neoplasm-bearing tissues. (**B**) Detection of CD34 (depicted in red)/Mannose (depicted in green) expression levels within neoplasm-bearing tissues. The *P*-value is less than 0.05.

The process involved assessing the abundance of M2-type macrophage markers and quantifying the vascularization within the tumor specimens from both sets of subjects via the technique of immunofluorescence. The Mannose Receptor, known colloquially as CD206, is a molecule heavily adorned with carbohydrates and is anchored to the membrane, predominantly found on the surface of macrophages, dendritic cells, and various endothelial cells that are not vascular in nature. It serves as an identifying marker for M2 macrophages. In addition to this, the antigen CD34 is present on the surface of a variety of cell lines, most notably the hematopoietic stem/progenitor cells across humans and other mammalian species, associating with the sialomucin family of proteins. CD34 also tags progenitor cells localized within diverse tissue types, inclusive of but not limited to the blood, the connective tissue, fibro-adipose progenitors, skeletal muscle, epidermal layers, vascular endothelium, and the epidermal lining of the dermis. Moreover, CD34 is prevalent in the mature vascular endothelial cells. The CD68 antigen is another marker employed for identifying cells within the mononuclear phagocyte system, namely monocytes and macrophages. In liver carcinomas, CD68 is typically used to denote the macrophages that have infiltrated the tumor. Macrophages signify a critical element within the tumor microenvironment, influencing the proliferation, dissemination, and therapeutic response of the neoplasms. An evaluation of cell counts positive for both CD68 and CD206 revealed a conspicuous elevation in the G-CSF-treated specimens over the control group. Additionally, the expression levels of CD34 together with CD206 within the G-CSF cohort were notably increased (see Figure 2A, 2B respectively). From these outcomes, one may deduce that G-CSF catalyzes the M2 polarization of macrophages associated with tumors, alongside augmenting angiogenesis.

### G-CSF augments tumor growth by stimulating angiogenic gene expression

**Figure 3:**
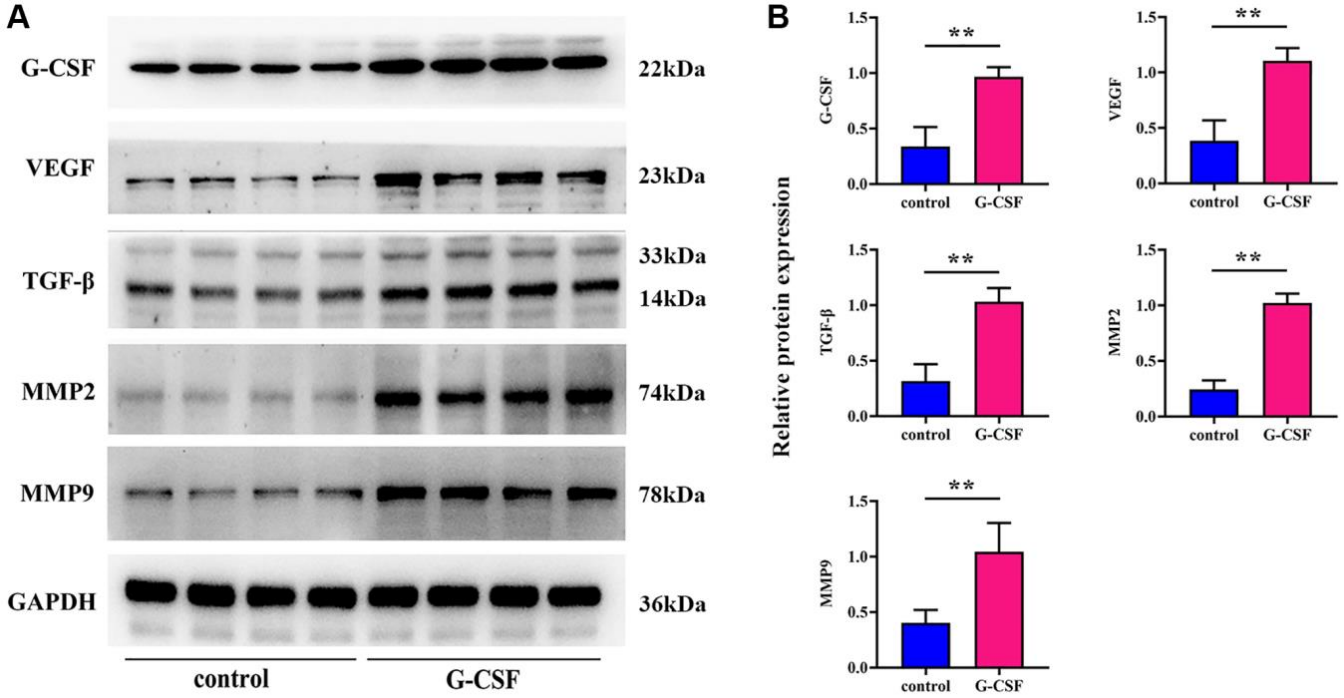
**Examination of G-CSF influence on SHP-2 and PI3K/AKT/mTOR pathway activity in neoplastic tissues utilizing western blotting techniques.** (**A**) Protein bands demonstrating SHP-2, phosphorylated PI3K, total PI3K, phosphorylated AKT, total AKT, phosphorylated mTOR, and total mTOR. (**B**) Comparative proteinic expression levels of SHP-2, the ratio of phosphorylated to total PI3K, the ratio of phosphorylated to total AKT, and the ratio of phosphorylated to total mTOR. The *P*-value is less than 0.05.

Assays employing Western Blot methods were executed to discern the expression levels of G-CSF, Vascular Endothelial Growth Factor (VEGF), Transforming Growth Factor-beta (TGF-β), and a suite of Matrix Metalloproteinases (MMPs) within the different sets of tumor samples. Initially, the modeling success rate of G-CSF was assessed in tandem with the examination of other molecules including VEGF and MMPs. The Western blotting results brought to light that the expression levels of G-CSF, VEGF, TGF-β, MMP2, and MMP9 proteins in the G-CSF treated group were significantly elevated relative to the control group (refer to Figure 3).

### G-CSF promotes tumor progression via SHP2-mediated enhancement of angiogenic gene expression

**Figure 4:**
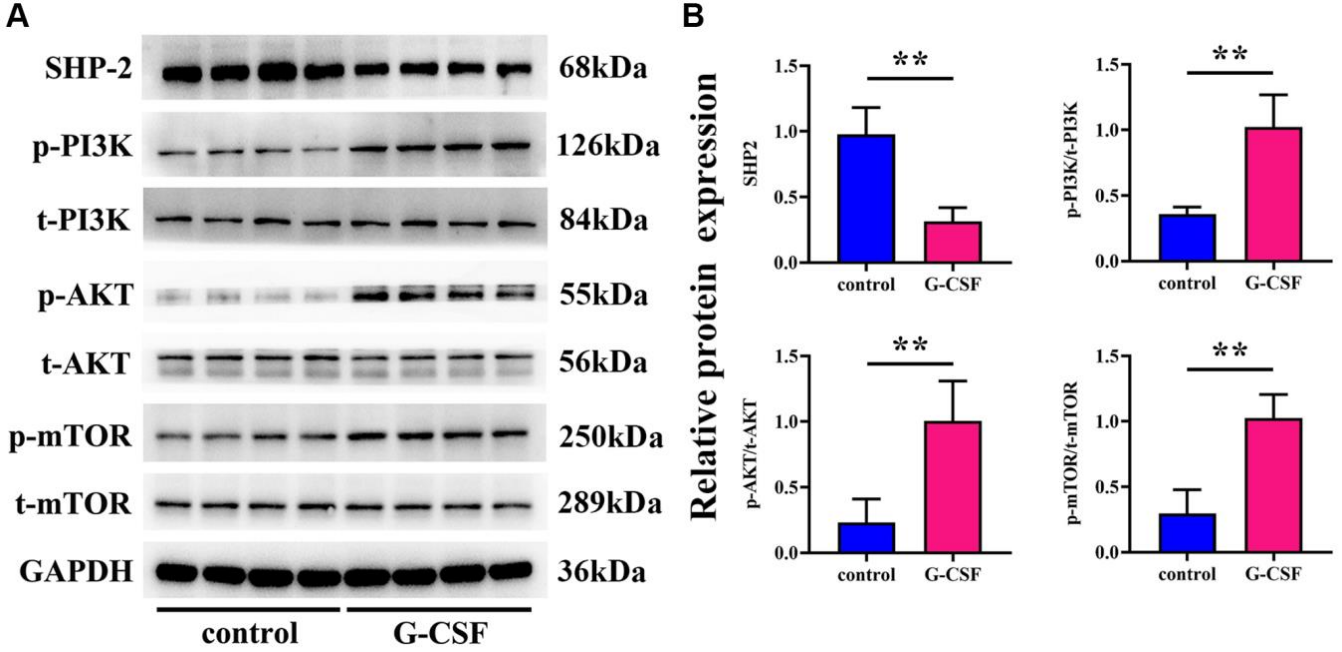
**Profiling of G-CSF, VEGF, TGF-β, and MMPs expression levels via western blotting techniques.** (**A**) Protein bands indicative of G-CSF, VEGF, TGF-β, and MMPs presence. (**B**) Comparative proteinic expression levels of G-CSF, VEGF, TGF-β, and MMPs. The *P*-value is less than 0.05.

Investigations on the activity of Src Homology 2 domain-containing phosphatase-2 (SHP-2) and the PI3K/AKT/mTOR signaling cascade within the tumor tissues were also conducted through Western Blot analysis. Former studies have reported that macrophage-associated SHP2 can impede the development of various cancers by attenuating signals from phosphorylated PI3K and AKT pathways. Consequently, we explored whether these mechanisms bore relevance under our experimental paradigm in the context of liver cancer progression. The outcomes of the Western blotting revealed a substantial reduction in SHP2 expression in the G-CSF group, with a concomitant rise in the expressions of phosphorylated PI3K, AKT, and mTOR, when compared to the control group. These observations propose that G-CSF may suppress SHP2 expression within tumor tissues, thereby facilitating the activation of the PI3K/AKT/mTOR signaling route (illustrated in Figure 4).

### G-CSF induces M2 macrophage formation in Co-culture with hepatocellular carcinoma cells

**Figure 5:**
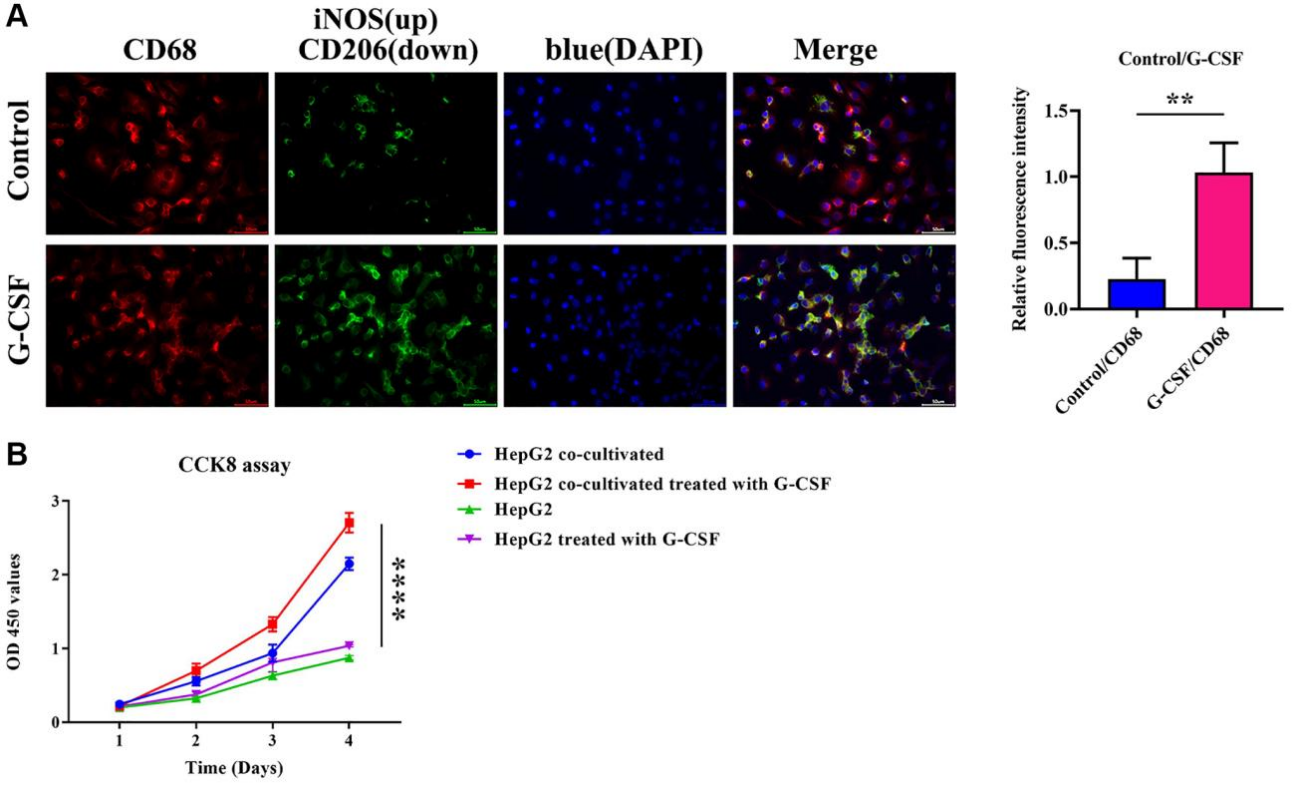
**Immunofluorescent determination of iNOS (depicted in green)/CD68 (depicted in red) and mannose (depicted in green)/CD68 (depicted in red) expression in macrophages.** (**A**) Immunofluorescence description of iNOS and mannose and quantitative analysis of the relative immunofluorescence intensities of iNOS and mannose. (**B**) Evaluation of the proliferative ability of HepG2 cells co-cultured with CCK8 detection. The *P*-value is less than 0.05.

Immunofluorescence methods were utilized to ascertain the expression levels of inducible Nitric Oxide Synthase (iNOS)/CD68 and Mannose/CD68 in macrophages. M1-type macrophages, which are a subset of immunologically activated macrophages, typically express iNOS as induced by cytokines such as Interferon-gamma (IFN-γ). iNOS is a catalyst in the production of substantial quantities of nitric oxide within M1-polarized macrophages, while CD206 is generally considered an indicator of the M2 macrophage subset. Following the extraction of macrophage-conditioned media, the immunofluorescence detected the expression of Mannose and iNOS in adherent cells. The iNOS/CD68 reading in THP-1 cells that underwent G-CSF treatment was relatively subdued, whereas the mannose/CD68 metric was notably elevated (as shown in Figure 5A). The solitary-cultured HepG2 cells exhibit a modest augmentation in proliferative capacity after G-CSF stimulation, as delineated in Figure 5B. Furthermore, when co-cultivated with THP1 cells, this enhancement in cell proliferation becomes notably more pronounced following exposure to G-CSF (as shown in Figure 5B). These data indicate that G-CSF potentially promotes M2 polarization while concurrently inhibiting M1 polarization within macrophages.

### G-CSF’s role in hepatocellular carcinoma progression: mediation by the PI3K/AKT/mTOR pathway and SHP2

**Figure 6:**
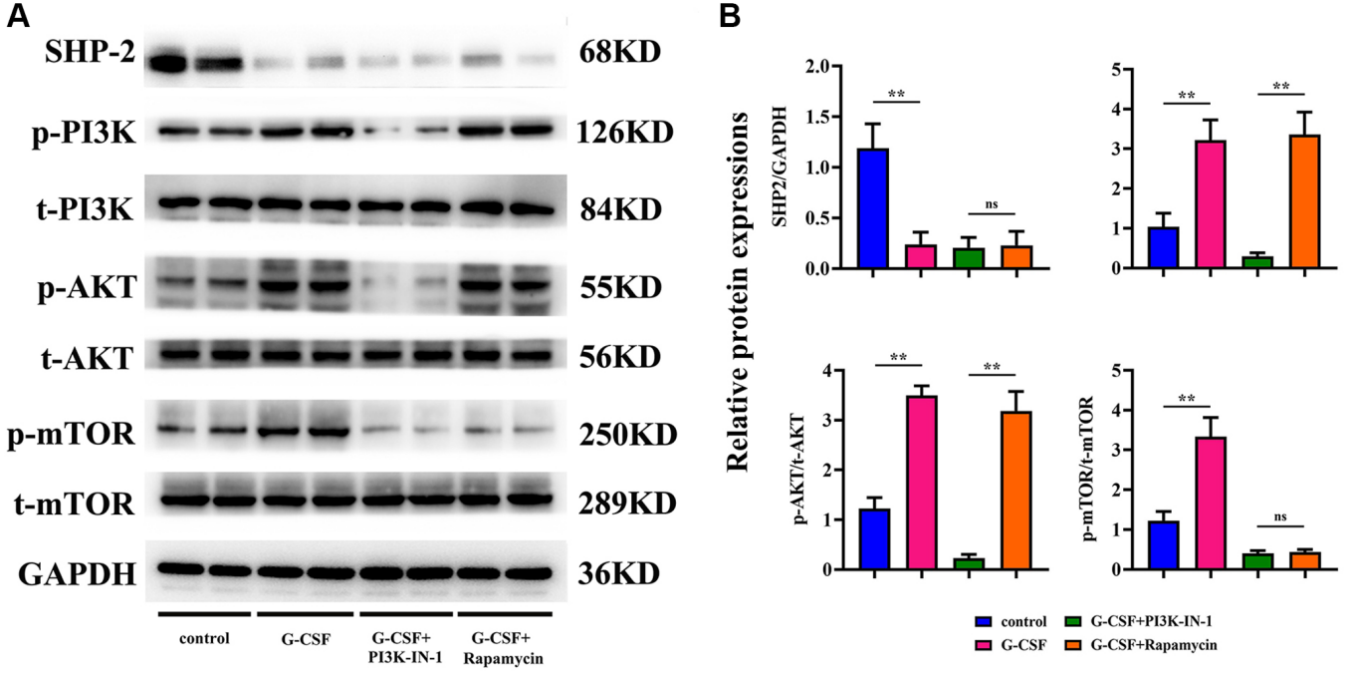
**Elucidation of SHP2 and PI3K/AKT/mTOR pathway proteinic levels in macrophages across experimental cohorts via western blotting techniques.** (**A**) Protein bands representing SHP-2, phosphorylated PI3K, total PI3K, phosphorylated AKT, total AKT, phosphorylated mTOR, and total mTOR. (**B**) Comparative proteinic expression levels of SHP-2, the ratio of phosphorylated to total PI3K, the ratio of phosphorylated to total AKT, and the ratio of phosphorylated to total mTOR. *P*-value is less than 0.05; *P*-value is greater than 0.05.

Further assessments via Western Blot analysis were performed to gauge the levels of proteins associated with the PI3K/AKT/mTOR pathway within the macrophages from each group under study. PI3K-IN-1 (XL-147 derivative 1) is a potent PI3K inhibitor, capable of interrupting the PI3K/Akt signaling axis. Rapamycin, on the other hand, exerts its antineoplastic effects on hepatic carcinoma cells by suppressing the mTOR signaling pathway, thereby influencing cell proliferation, growth, and metabolism. Analyzing the protein expression through Western blotting, a discernible diminishment in SHP2 levels was observed in macrophages treated with G-CSF, along with a marked amplification in the relative expressions of phosphorylated PI3K/tot-PI3K, AKT/tot-AKT, and mTOR/tot-mTOR, as opposed to the control group. However, introducing PI3K-IN-1 or rapamycin to the G-CSF-treated groups resulted in no significant deviation in SHP2 expression levels, when benchmarked against the singular G-CSF treatment. Comparatively, the expressions of phosphorylated PI3K, AKT, and mTOR exhibited a substantial decline in the group treated with G-CSF and PI3K-IN-1, while only the phosphorylated mTOR readings underwent a notable drop in the group that integrated the usage of G-CSF and rapamycin (depicted in Figure 6). Such evidence suggests that G-CSF primes the PI3K/AKT/mTOR signaling pathway by curtailing SHP2 expression in the tumor-affiliated macrophages.

### G-CSF promotes angiogenesis in VEC4-10 cells through PI3K/AKT/mTOR pathway activation

**Figure 7:**
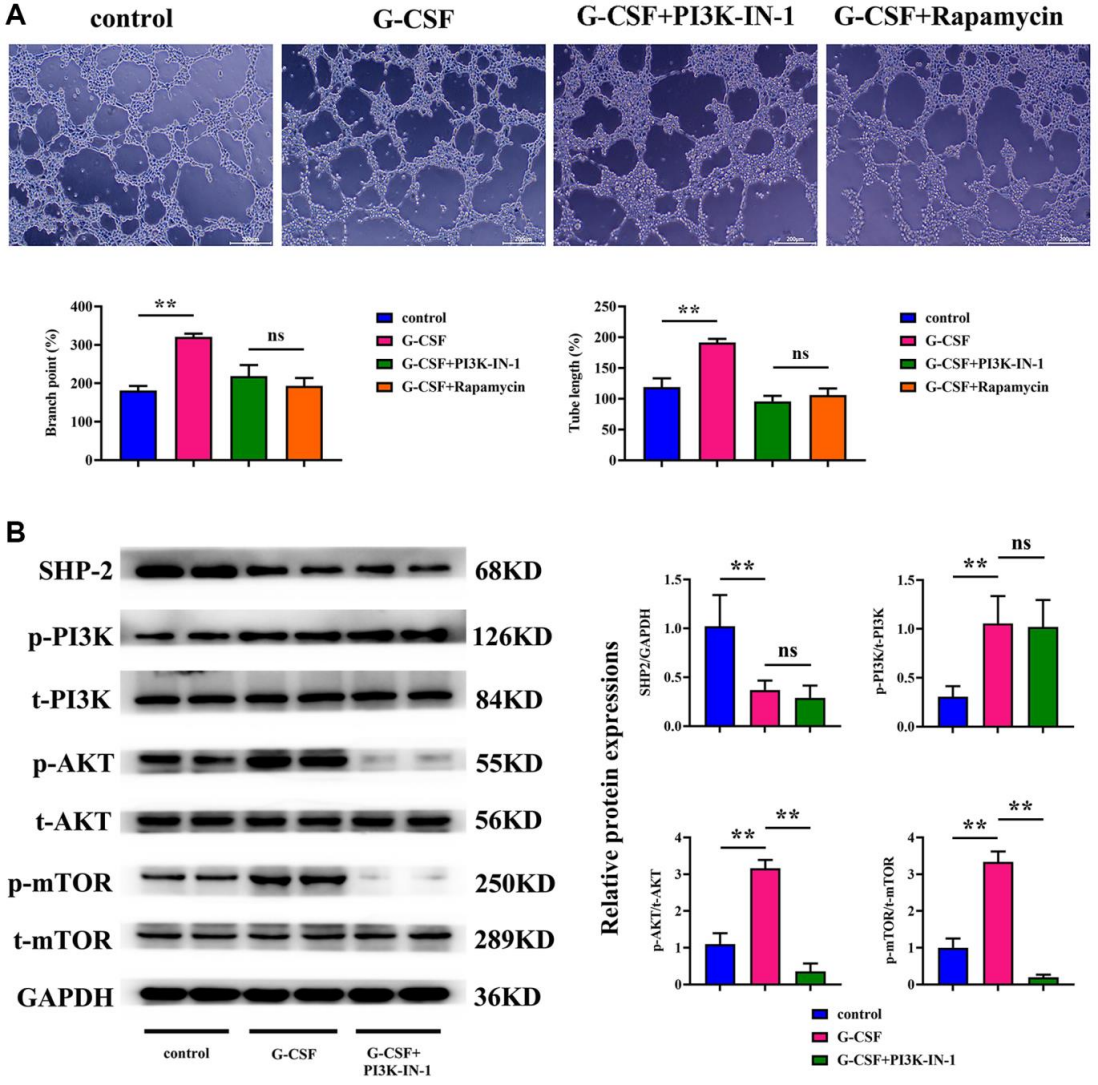
**Assessment of the angiogenic potential of SVEC4-10 cells.** (**A**) Conclusions drawn from angiogenesis assays. (**B**) Statistical enumeration of angiogenic instances. *P*-value is less than 0.05; *P*-value is greater than 0.05.

Lastly, the capillary formation capabilities of SVEC4-10 cells were scrutinized. The angiogenesis assays exhibited a significant surge in both the count and length of vasculature in the G-CSF-treated group over the control cluster. However, upon the introduction of PI3K-IN-1 or rapamycin to the G-CSF regimen, a pronounced reduction in these vascular metrics was observed. No substantial discrepancy was noted in the vascular count or length between the groups treated with G-CSF and rapamycin in comparison to the G-CSF and PI3K-IN-1 cohort (as illustrated in Figure 7). This indicates that G-CSF promotes the expansion of hepatocellular carcinoma cells and their angiogenic capabilities by repressing SHP2 function within the tumor-associated macrophages, which in turn triggers the PI3K/AKT/mTOR signaling pathway.

## DISCUSSION

In recent epochs, academia has cast an intensified gaze on the tumor microenvironment's role within hepatic carcinoma, with predominant investigations focusing on the multifaceted microenvironments, such as the inflammatory, immunomodulatory, angiogenetic, intestinal, and hepatic regenerative niches referenced in the literature [13]. The pronounced heterogeneity of hepatic malignancies, coupled with the current diagnostic and therapeutic modalities' confined efficacy, underscores the imperative of delving into the molecular underpinnings governing the inception and progression of hepatocellular carcinoma (HCC), in pursuit of novel therapeutic targets.

Granulocyte Colony-Stimulating Factor (G-CSF), an indispensable harbinger in the maturation of granulocytes, assumes a pivotal role in the proliferation and lineage specification of neutrophils. Representing a cardinal hematopoietic molecule, it orchestrates the transformation of bone marrow progenitors into neutrophils. Tumor-associated macrophages (TAMs) hold sway as a primary constituent of immune infiltrates within the solid tumor stroma; their presence—potentially up to 50% within neoplastic tissues—demarcates their vital functionality in oncogenesis and tumor progression [14]. A cadre of scholarly works elucidates TAM’s capacity to modulate neoplastic invasion, metastasis, angiogenesis, and lymphangiogenesis [15]. Emerging evidence posits that G-CSF can exert an influence upon human monocytes, modulating cytokine secretion, and abetting the transition of macrophages towards an M2 phenotype. In light of this, our research employed a BALB/c mouse model harboring an in-situ hepatic tumor to scrutinize the impact of G-CSF on M2 macrophages and endothelial cells within the HCC microenvironment, to fathom G-CSF's role in the vascular reconfiguration of HCC.

Presently, the discourse on G-CSF predominantly intersects with classical inflammatory cascades. For instance, pediatric patients suffering from muscular dystrophy evidenced augmented CD163 expression in monocytes upon G-CSF stimulation [16], signaling the cytokine’s propensity to favor monocyte skewing towards an M2 macrophage phenotype. Further research argues that G-CSF invocation can dampen LPS-elicited cytokine release and directly stifle monocytes’ pro-inflammatory factor production whilst amplifying IL-10 secretion [17, 18], suggesting G-CSF's agency in fostering M2 inflammation. From these findings, one might deduce G-CSF’s role in monocyte polarization towards M2 macrophages and in bolstering M2-type inflammation, yet querying whether such a mechanism transpires similarly within neoplastic contexts warrants additional inquiry.

Given the immunosuppressive signature of TAMs, epitomized by M2 macrophages, it is well-acknowledged that these cells are prolific expressers of Mannose. Our research utilized immunofluorescence techniques to gauge Mannose/CD68 and CD34/Mannose expression within tumor cohorts and appraised iNOS/CD68 and Mannose/CD68 expressions in macrophages. Experimental observations revealed an aggregation of M2 macrophages in murine hepatic tissue administered G-CSF *in situ*, in stark contrast to the sparse M2 macrophage presence in untreated tumors. Thus, we posit that G-CSF facilitates M2 polarization while concurrently thwarting M1 macrophage polarization.

Encoded by the Ptpn11 gene, SHP-2—a ubiquitously manifested protein tyrosine phosphatase—anchors pivotal functions regarding survival, proliferation, and differentiation across distinct cellular entities [19]. Its ubiquity within both neoplastic and immune cells has stirred conjectures over its duality in either advancing or impeding tumorigenesis. Pertinent to hepatic oncology, the absence of SHP-2 has been linked to liver inflammatory states and necrosis, potentiating nodule hyperplasia and bolstering inflammatory messaging, potentially catalyzing hepatic tumorigenesis [20, 21].

SHP2’s contribution to the activity of the PI3K/AKT/mTOR signaling axis stems from interactions with various receptor tyrosine kinases and kinase proteins, influencing their catalytic functions and attendant signaling pathways, including those engaged with the PI3K/AKT/mTOR route. Studies suggest that SHP2 inhibition can stunt this signaling axis, potentially arresting cell cycles, spurring apoptosis, and mitigating cellular proliferation, ultimately exerting a suppressive impact on tumor growth and survival. Such results echo our findings, where SHP-2 expression in G-CSF-stimulated THP-1 cells appeared markedly diminished, while phosphorylated derivatives of PI3K, AKT, and mTOR surged in expression relative to their total proteins, signifying G-CSF's promotion of PI3K/AKT/mTOR signaling through SHP-2 inhibition.

Vascular Endothelial Growth Factor (VEGF) has achieved notoriety as a vascular permeability factor and an endothelial cell-centric mitogen, fostering vascular permeability augmentation, extracellular matrix degradation, endothelial migration, proliferation, and angiogenesis. Conversely, Transforming Growth Factor β (TGF-β) emerges as a versatile cytokine within the TGF superfamily, containing intrinsic growth-inhibitory proteins. Its escalated expression frequently aligns with heightened malignancy across various cancers, alongside aberrations in cellular responses to TGF-β's growth-inhibitory cues—its immunosuppressive functions, therefore, assume prominence in tumorigenesis. Anomalous immune suppression is also implicated in the pathophysiology of autoimmune diseases, with effects substantially contingent upon the milieu of co-present cytokines. Matrix metalloproteinases (MMPs), collagens' denaturing enzymes, and tadpole tail resorption agents form a faction within the metzincins superfamily—an assemblage of zinc-dependent endopeptidases—that catalyze the degradation of several extracellular matrix (ECM) proteins. MMPs engage in interplays with vascular smooth muscle growth, proliferation, migration, and relaxation; these interactions bear relevance to endothelial function, angiogenesis, apoptosis, tissue repair, wound healing, gestational embryo implantation, and trophoblast invasion. Within the angiogenetic process, VEGF and Ang-2 are essentially pivotal elements fostering neovascular growth, urging endothelial cell proliferation and angiogenesis [22]. MMPs orchestrate ECM non-polysaccharide refractors' dissolution [23], advancing the remodeling of the neoplastic ECM and participating in angiogenesis. Its clinical prognosis evaluation and treatment is a major research direction. Ferroptosis and cuproptosis are new forms of cell death, thought to be important factors in cancer progression. [24] When VEGF, MMPs, and TGF-β concert their efforts, they can disrupt the equilibrium and homeostasis of existing vasculature within neoplasms, augmenting the germination and anastomosis of blood vessels, therewith engendering a favorable milieu for neovascularization within tumors. Our investigations uncovered that G-CSF treatment markedly upregulated VEGF, TGF-β, and MMP expression levels within neoplastic tissues, accompanied by a concomitant rise in the CD34 endothelial cell marker, positing that G-CSF can indeed abet the genesis of vascular endothelial cells within tumors, potentially propelling ECM remodeling and vessel formation.

In our comprehensive exploration, the Granulocyte Colony-Stimulating Factor (G-CSF) has been implicated as a significant contributor to the progression of hepatocellular carcinoma. Empirical evidence derived from studies conducted on nude mice bearing such tumors points to an undeniable exacerbation of tumor growth upon the administration of G-CSF. Specifically, G-CSF treatments yielded an appreciable expansion in tumor size relative to their counterparts without such intervention. These observations highlight the deleterious potential of G-CSF in fostering not only the occurrence but remarkably the expansion of hepatocellular carcinomas. Delving deeper into the underlying mechanisms, we observed a distinct tendency for G-CSF to induce polarization of macrophages into the M2 phenotype, known to accommodate tumor progression. This polarization was evidenced through the elevation of M2-type macrophage markers as indicated by immunofluorescence analyses of the tumor samples. The presence of molecular identifiers such as CD206, a mannosylated receptor highly prevalent on macrophages, and CD34—a lineage marker for progenitor cells and vascular endothelium—were found to be considerably upregulated in G-CSF-treated organisms. Simultaneously, a substantial surge in both CD206 and CD34 expression levels was indicative of increased angiogenesis, respectively. This engendered the hypothesis that G-CSF catalyzes the M2 macrophage polarization, concomitantly promoting the formation of new blood vessels within the tumor matrix. Furthermore, our investigatory efforts shed light on the potential of G-CSF to invigorate tumor proliferation through the stimulation of angiogenic gene expression. By employing Western Blot methodologies, we ascertained significant enhancements in the expression levels of G-CSF, Vascular Endothelial Growth Factor (VEGF), Transforming Growth Factor-beta (TGF-β), as well as a panel of Matrix Metalloproteinases (MMPs) among the tumors exposed to G-CSF, revealed in Figure 3. This observation suggests an inherent capacity of G-CSF to augment tumorigenesis via enhanced angiogenic signaling. Additionally, the G-CSF-facilitated tumor progression could potentially be intermediated by an SHP2-regulated escalation of angiogenic gene expression. Investigations regarding the activity of Src Homology 2 domain-containing phosphatase-2 (SHP-2) and the consequential effects on the PI3K/AKT/mTOR signaling cascade in tumor specimens demonstrated a notable suppression of SHP2 allied with an activated state of the aforementioned pathways. Such findings propose that the suppression of SHP2 within tumor tissues may be a stratagem through which G-CSF endorses the initiation of the PI3K/AKT/mTOR pathway, a critical hallmark in the advancement of liver cancer. In macrophage and hepatocellular carcinoma cell co-cultures, G-CSF has also been revealed to skew macrophage differentiation favorably toward an M2 phenotype. Here, using immunofluorescence, we documented the diminished levels of inducible Nitric Oxide Synthase (iNOS) in favor of an upregulated mannose-associated M2 profile. This supports the tenet that G-CSF selectively promotes an environment conducive to M2 macrophage polarization, thereby undermining the M1 phenotype which is typically antagonistic to cancer progression. The undeniable interplay between G-CSF and the pivotal PI3K/AKT/mTOR pathway within macrophages was further corroborated by Western Blot analyses. The inhibition of PI3K and mTOR using PI3K-IN-1 and Rapamycin, respectively, illustrated that the presence of G-CSF significantly attenuates SHP2 expression while correspondingly enhancing activation signals along PI3K/AKT/mTOR axis, though no noticeable alteration in SHP2 levels occurred upon co-treatment with G-CSF and these inhibitors. This evidence strongly reinforces the concept that G-CSF primes hepatocellular carcinoma progression by modulating intracellular signals that favor tumor growth and angiogenesis. Lastly, the angiogenic potential of SVEC4-10 cells in response to G-CSF was appraised, signifying a substantive proliferation in both the count and extension of capillary structures upon G-CSF treatment. However, the integration of PI3K-IN-1 or Rapamycin to the therapeutic regimen exhibited a sensible reduction in these vascular indices, indicating that the pro-angiogenic effects of G-CSF are indeed modulated via the PI3K/AKT/mTOR pathway and attenuated SHP2 function within the tumor-nexus macrophages. In summation, our investigative foray illuminates the intricate role of G-CSF in advancing hepatocellular carcinoma progression, explicating a comprehensive mechanistic schema wherein G-CSF potentiates macrophage polarization towards a tumor-friendly M2 phenotype, instigates gene expression conducive to angiogenesis, and engages pivotal intracellular pathways that bolster the tenuous bridge between cancer proliferation and vascular development.

## CONCLUSION

In summary, G-CSF can activate the PI3K/AKT/mTOR signaling pathway by inhibiting SHP2 activation in tumor-associated macrophages, and promoting the proliferation, migration, and angiogenesis of hepatocellular carcinoma cells, leading to the progression of hepatocellular carcinoma.
